# Cloning and preliminary verification of telomere-associated sequences in upland cotton

**DOI:** 10.3897/CompCytogen.v14i2.49391

**Published:** 2020-04-06

**Authors:** Yuling Liu, Zhen Liu, Yangyang Wei, Yanjun Wang, Jiaran Shuang, Renhai Peng

**Affiliations:** 1 Anyang Institute of Technology, Anyang, Henan, 455000, China Anyang Institute of Technology Anyang City China

**Keywords:** *G. hirsutum*, telomere-associated sequence, cloning, FISH

## Abstract

Telomeres are structures enriched in repetitive sequences at the end of chromosomes. In this study, using the telomere primer AA(CCCTAAA)_3_CCC for the single primer PCR, two DNA sequences were obtained from *Gossypium hirsutum* (Linnaeus, 1753) accession (acc.) TM-1. Sequence analysis showed that the two obtained sequences were all rich in A/T base, which was consistent with the characteristic of the telomere-associated sequence (TAS). They were designated as GhTAS1 and GhTAS2 respectively. GhTAS1 is 489 bp long, with 57.6% of A/T, and GhTAS2 is 539 bp long, with 63.9% of A/T. Fluorescence *in situ* hybridization results showed that both of the cloned TASs were located at the ends of the partial chromosomes of *G. hirsutum*, with the strong signals, which further confirmed that GhTAS1 and GhTAS2 were telomere-associated sequences including highly tandemly repetitive sequences. Results of blast against the assembled genome of *G. hirsutum* showed that GhTAS sequences may be missed on some assembled chromosomes. The results provide important evidence for the evaluation of the integrity of assembled chromosome end sequences, and will also contribute to the further perfection of the draft genomes of cotton.

## Introduction

Telomeres are DNA-protein complexes at the ends of chromosomes (Blackburn 1991). Telomere structures are highly conserved, and vary surprisingly little between organisms (Richards and Ausubel 1988, Ganal et al. 1991, Fajkus et al. 2005, Watson and Riha 2010). In humans, telomere repeated sequences are composed of conserved a minisatellite sequence unit 5’-TTAGGG-3’ (Moyzis et al. 1988), whereas in *Tetrahymena* (Furgason, 1940) each chromosome end has a conserved 5’-TTGGGG-3’ telomere repeat unit (Blackburn and Gall 1978). The first plant telomere DNA sequence, 5’-TTTAGGG-3’ tandem repeat, was isolated from *Arabidopsis thaliana* (Linnaeus, 1753) (Richards and Ausubel 1988). Subsequent studies have demonstrated that the *Arabidopsis*-type telomeres presented in most plants (Fajkus et al. 2005, Ling et al. 2012, Schrumpfová et al. 2019). At the same time, other studies have shown that some plants lacked typical telomere tandem repeat 5’-TTTAGGG-3’, which sheds more light on telomere function and how telomeres responded to genetic change (Adams et al. 2001, Sýkorováet al. 2003a, Peška et al. 2015).

Telomere tandem repeats located at the end of chromosomes represent only a part of the end of chromosomes. Telomere-associated sequences (TASs) located directly proximal to telomere tandem repeats (Li et al. 2009) play an important role in telomere maintenance and chromosome stability through epigenetic modification or recombination (Cross et al. 1990, Zhong et al. 1998, Sýkorováet al. 2003b, Tran et al. 2015). In addition, TAS is also a good marker at the end of the genetic linkage map. Three TASs cloned from rice showed high polymorphism at the ends of chromosome arms of different rice varieties based on the results of genetic mapping (Ashikawa et al. 1994). Despite functional importance, the nucleotide sequences in the subtelomere region have not been fully resolved in many sequenced genomes (Lese et al. 1999, Mefford and Trask 2002, Mizuno et al. 2006). So, more work is needed to reveal the structure and function of the subtelomeres.

At present, there is relatively little research on cotton telomere. Combining FISH using the *Arabidopsis*-type telomere sequence amplified from *Arabidopsis* genomic DNA and BAL-31 digestion, Ling et al. (2012) published the first study on cotton telomeres, which proved the *Arabidopsis*-type telomere sequence existed in the cotton genome. *G. hirsutum* is the most important cultivated cotton species. So far, different versions of the genome sequence have been released (Li et al. 2015, Zhang et al. 2015, Wang et al. 2019, Hu et al. 2019), however, high content of repetitive sequences affects the quality of genome assembly (Sýkorová et al. 2013, Liu et al. 2016). TAS occupies a large proportion in subtelomere tandem repeats regions. Therefore, in order to improve the quality of genome assembly, nucleotide sequences in the subtelomere region need to be further analyzed.

## Material and methods

### Plant materials

The plant material was *G. hirsutum* acc. TM-1 (AADD)_1_, which was planted in the experimental field of Anyang Institute of Technology, Henan, China. Genomic DNA was isolated from fresh young leaves using the modified CTAB method (Song et al. 1998). Root tip material used for *G. hirsutum* chromosome preparation were harvested from the about 6-day seedlings planted in an incubator and pretreated by 25 ppm cycloheximide at 20 °C for 80 min, then fixed in methanol-acetic acid (3:1) and stored at 4 °C for 24 h. Squashes of root tips were prepared according to Liu et al. (2017).

### Primers

The eight single primers of the plant telomere repeat were selected from NCBI database (https://www.ncbi.nlm.nih.gov) according to the previous studies for single primer PCR (Burr et al. 1992, Gong et al. 1998, Weiss-Schneeweiss et al. 2004, Liu et al. 2005). The primers sequence information is shown in Table 1.

### Cloning and sequencing of telomere-associated sequences

The selected single primers of the plant telomere repeat sequence (Table 1) were amplified by single primer PCR using the genomic DNA of *G. hirsutum* as template, to find the suitable conditions for obtaining promising products and candidates for subtelomeric regions. The amplification procedure was as 95 °C for 3 min, followed by 35 cycles of 95 °C for 15 s, 55 °C/60 °C for 15 s, 72 °C for 30 s, and a final extension at 72 °C for 5 min. The amplification products were detected by 1% agarose gel electrophoresis, and the appropriate single primer and annealing temperature were selected based on the above result. Then, PCR amplification was performed using the selected single primer in a 50 μl reaction volume containing 25 μl of 2 × Phanta Max Buffer, 1 μl of Phanta Max Super-Fidelity DNA Polymerase (Vazyme), 0.8 μmol/L of the telomeric single primer, and 10 ng of genomic DNA. The objective band from PCR was recovered by gel extraction kit (SanPrep Column DNA Gel Extraction kit, Sangon Biotech) and was cloned into *Trans*1-T1 competent cells by the *pEasy*-Blunt Simple Cloning Vector (TransGen Biotech) according to the manufacturer´s instructions. The positive clones were selected for sequencing by Shanghai Sangon.

**Table 1.** Telomere primer sequence information.

**Name**	**Taxonomic name**	**Reference**	**Sequence**
TR1	*Oryza sativa* (Linnaeus, 1753)	Gong et al. 1998	(TTTAGGG)_3_
TR2	*Zea mays* (Linnaeus, 1753)	Burr et al. 1992	(TTTAGGG)_4_
TR3	*Othocallis siberica* (Linnaeus, 1753)	Weiss-Schneeweiss et al. 2004	(TTTAGGG)_5_
TR4	*Ginkgo biloba* (Linnaeus, 1771)	Liu et al. 2005	(CCCTAAA)_3_
TR5	*Brassica campestris* (Linnaeus, 1753)	Kapila et al. 1996	(CCCTAAA)_3_CCC
TR6	*Othocallis siberica*	Weiss-Schneeweiss et al. 2004	AA (CCCTAAA)_3_CCC
TR7	*Zea mays*	Burr et al. 1992	(CCCTAAA)_4_
TR8	*Othocallis siberica*	Weiss-Schneeweiss et al. 2004	(CCCTAAA)_5_

### Software and websites for sequences analysis

DNAMAN software was used for extraction and alignment of cloned sequences. Repetitive sequence analysis was performed using the online program CENSOR (https://www.girinst.org/censor/index.php). BLAST algorithm blastn (https://www.cottongen.org/blast) was used to identify GhTAS from *G. hirsutum* genome database (*Gossypium hirsutum* ZJU v2.1, a1) (Hu et al. 2019). All the above analyses were performed according to the default parameters.

### FISH validation

The TAS plasmid DNA was extracted using the TIANprep Mini Plasmid Kit according to the instructions. Then, TAS plasmid DNA was labeled with DIG-Nick Translation Mix (Roche). The 45S rDNA probes derived from *Arabidopsis thaliana* (Gan et al. 2013) were labeled with biotin-Nick Translation Mix (Roche) according to the instructions of the manufacturer. Chromosome preparation and FISH were performed according to the previous methods (Liu et al. 2017).

## Results

### Optimization of the single primer PCR

**Figure 1.**
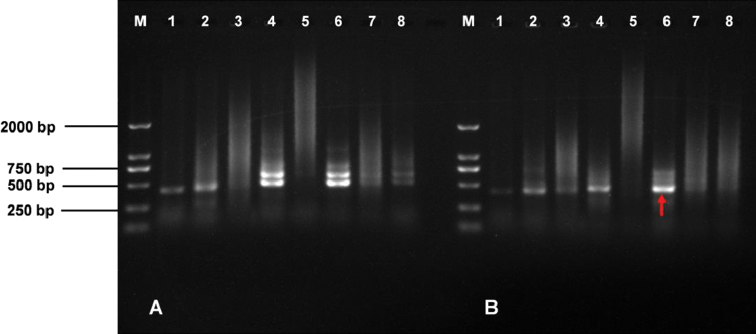
Amplification results of candidate single primers. **M** Marker **A** and **B** the annealing temperature is 55 and 60 °C respectively **1–8** primers TR1–TR8.

According to the melting temperature (TM) value distribution of the eight candidate single primers (55 °C–62 °C), two annealing temperatures were selected, namely 55 °C and 60 °C. The results of PCR amplification showed that an obvious band of roughly 500 bp was amplified using the single primer TR6 under the two annealing temperatures, especially, the band amplified under annealing temperature of 60 °C showed better specificity and higher brightness (Fig. 1B–6). So, the primer TR6 (AA (CCCTAAA)_3_CCC) was chosen for the following PCR amplification.

### Cloning of TAS

**Figure 2.**
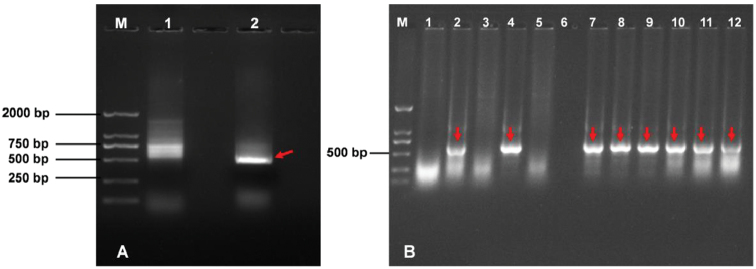
Results of cloning and positive test. **A** PCR amplification results **M** Marker **1** Common Taq enzyme **2** High-fidelity enzyme. **B** Positive test of bacterial colony PCR **M** Marker **1–12** the candidate clones.

A single band with a size of roughly 500 bp was amplified using the single primer TR6 under the annealing temperature of 60 °C with Phanta Max Super-Fidelity DNA Polymerase (Fig. 2A–2). After transformation, eight positive clones were obtained after a positive test from transformed clones (Fig. 2B). Then, the eight positive clones were sequenced.

### Sequences analysis

**Sequence component analysis**

**Figure 3.**
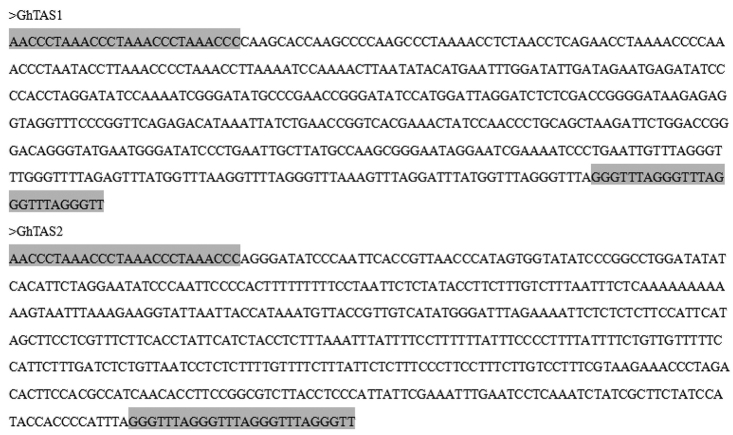
Sequences of the two TASs. The grey shadows are reverse complementary sequences of the telomere primer TR6.

Sequence analysis of the eight positive clones revealed that all clones had the same forward and inverted telomere primer sequence at the two ends. Sequence alignment showed that there were two different internal sequences in eight sequences. So, the two different cloned DNA sequences with different size of 488 bp and 538 bp were selected and named as GhTAS1 and GhTAS2 (Fig. 3). Their sequences had been uploaded to GenBank (accession No. MT078976 and MT078977).

The two sequences were rich in A/T bases, that is, 57.6% and 63.9% respectively. Repeat masking analysis indicated that the tandem repeats content were 31.35% in GhTAS1 and 42.38% in GhTAS2, which mainly consisted of satellite DNA and transposable elements. The above results are consistent with the typical characteristics of telomere-associated sequences (Li et al. 2009).

### Homology analysis of GhTASs

Sequence alignment results of DNAMAN shown that GhTAS1 and GhTAS2 had low homology, with the sequence similarity of 38.90%, which may be due to their different chromosomal sources.

After comprehensive comparison of the obtained TASs of *G. hirsutum* and the TASs of *Arabidopsis thaliana*, *Glycine* max (Linnaeus, 1753), *Oryza sativa* (Linnaeus, 1753), *Zea mays* (Linnaeus, 1753), *Larix gmelinii* (Ruprecht, 1920) listed on NCBI, it was found that their similarity was low, ranging from 25% to 50% (Table 2). All these indicated that the cloned telomere-associated sequences had obvious species specificity.

**Table 2.** Similarity of telomere-associated sequences between *G. hirsutum* and other plants.

Species	NCBI accession No.	TASs of *G. hirsutum*	
		GhTAS1	GhTAS2
*Arabidopsis thaliana*	AC074298.1	39.60%	36.71%
	AM177016.1	14.08%	12.94%
	AM177019.1	13.52%	13.93%
	AM177060.1	10.88%	10.15%
*Glycine* max	AF041999.1	20.24%	16.79%
*Oryza sativa*	U12056.1	28.71%	25.27%
*Zea mays*	S46927.1	48.70%	41.93%
*Larix gmelinii*	EF474441.1	31.40%	30.57%

### BLAST of GhTAS1 and GhTAS2 against *G. hirsutum* genome

**Figure 4.**
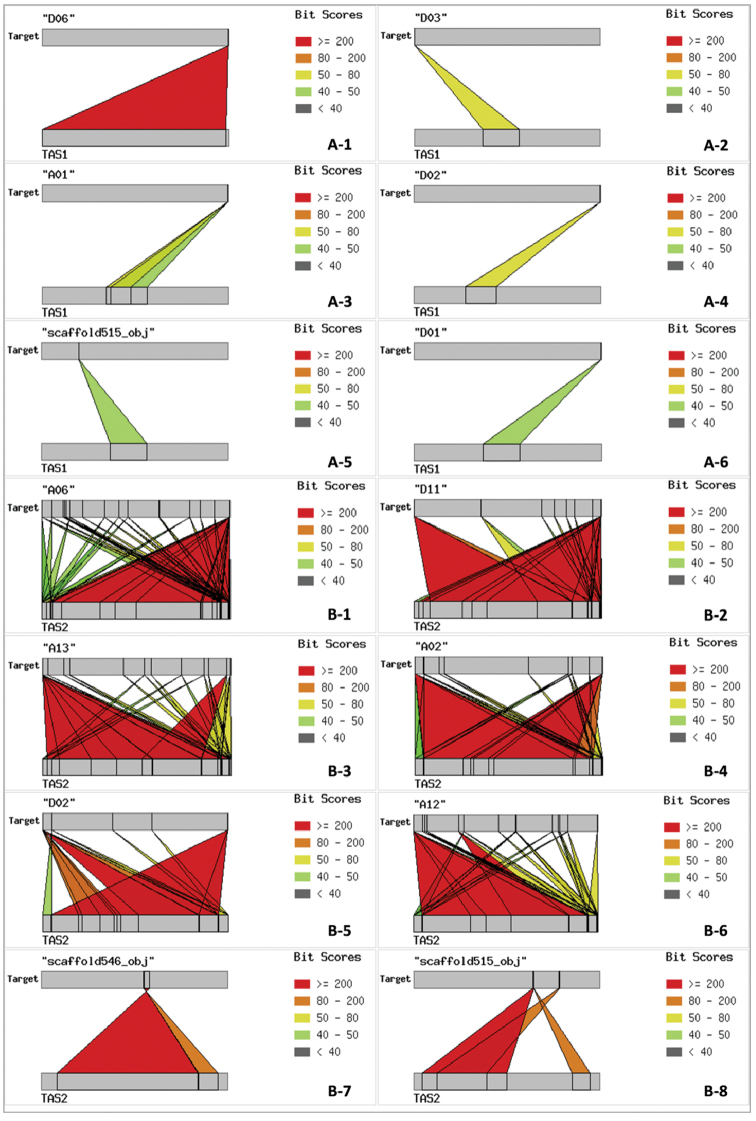
Localization patterns of GhTAS1 and GhTAS2 on *G. hirsutum* partial chromosomes. **A** GhTAS1 **B** GhTAS2.

GhTAS1 and GhTAS2 were found using blastn with the latest *G. hirsutum* genome sequence (*Gossypium hirsutum* ZJU v2.1, a1) in Cottongen (https://www.cottongen.org/). Results showed that GhTAS1 was mapped onto five chromosomes and one scaffold of *G. hirsutum*, and GhTAS2 was mapped onto all 26 chromosomes and 14 scaffolds of *G. hirsutum* with different E-value. The partial blast results with lower E-value were listed in Table 3. GhTAS1 was localized at one end of the chromosome D06, with a higher similarity of 98.98%, and was localized at the single end of chromosomes D03, A01, D02 and D01, as well as Scaffold515, with lower similarity (Fig. 4A). GhTAS2 showed higher chromosomes coverage than GhTAS1. Among the all blast results, GhTAS2 was localized at both ends of chromosomes D11, A13, A02 and D02 and at the single end of chromosomes A06, A12 and two scaffolds with higher similarity (Fig. 4B). At the same time, unlike GhTAS1, the GhTAS2 sequence is also mapped to other chromosomal regions in addition to the ends of chromosomes (Fig. 4B1–6).

### Chromosome localization of GhTAS1 and GhTAS2 based on FISH

**Figure 5.**
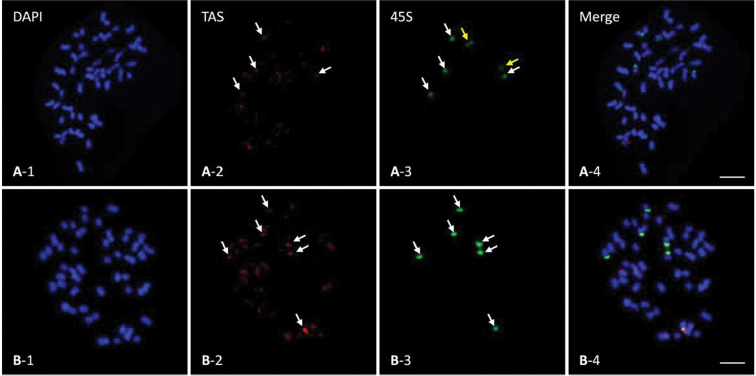
FISH on *G. hirsutum* chromosome with 45S rDNA (green) and GhTAS1 or GhTAS2 (red) probes, Scale bars: 5μm. The white arrows showed co-location **A** GhTAS1 **B** GhTAS2.

To examine the chromosome physical location of GhTAS1 and GhTAS2, we carried out FISH on *G. hirsutum* metaphase chromosomes using a digoxin-labeled GhTAS probe and a biotion-labeled 45S rDNA probe. The results showed that GhTAS1 had signals at the end of nearly half of the chromosomes of *G. hirsutum*, and most of them were distributed at the single end. The signal intensity on different chromosomes was also different (Fig. 5A–2, A-4). GhTAS2 has signals on both ends of most chromosomes of *G. hirsutum* (Fig. 5B–2, B-4). Three pairs of 45S rDNA signals were detected on the chromosomes of *G. hirsutum* (Fig. 5a–3 and 5B–3 arrows). Two pairs of GhTAS1 signals were collinear with 45S rDNA (Fig. 5A–2 arrows). Three pairs of GhTAS2 signals were collinear with 45S rDNA (Fig. 5B–2 arrows). In addition, the chromosomes carrying GhTAS2 FISH signals were much more than those with GhTAS1 FISH signals (Fig. 5A–2, B-2), which was similar to the blast results (Fig. 4, Table 3).

**Table 3.** Partial blast results of GhTAS1 and GhTAS2 in the *G. hirsutum* genome.

**Sequence name**	**Genomic location**	**Query matches**	**Hit matches**	**Identity (%)**
GhTAS1	D06	1–488	65407147–65406660	98.98%
	D03	184–281	26586–26683	78.57%
	A01	171–237	118151185–118151119	82.09%
	D02	138–219	69751633–69751551	79.52%
	Scaffold515-obj	184–281	9914–9817	75.51%
	D01	184–281	64676574–64676477	75.51%
GhTAS2	A06	14–537	126445179–126444656	99.62%
	D11	14–537	71336660–71336138	98.47%
	A13	14–535	47688–48202	94.08%
	A02	25–512	40084–39589	88.15%
	D02	25–512	69751559–69752073	86.68%
	A12	25–512	30186–29672	86.15%
	Scaffold546-obj	46–455	8556–8146	89.07%
	Scaffold515-obj	25–271	31264–31514	89.33%
	A09	25–315	83200103–83200398	86.96%
	A11	25–271	121355653–121355904	88.54%
	scaffold407_obj_A03	59–271	36503–36719	92.24%
	A07	25–271	96580716–96580969	88.24%
	D10	278–455	66830830–66831007	93.26
	A10	25–271	115081227–115081476	87.75%
	A05	285–455	39831434–39831267	93.60%
	A01	278–455	118169784–118169962	91.06%
	D08	278–512	69075939–69076196	84.11%
	D03	278–455	23313–23139	91.01%
	D09	278–442	51987281–51987445	91.52%

## Discussion

In this study, the telomere primer AA(CCCTAAA)_3_CCC was used as a single primer to obtain the TAS sequences of *G. hirsutum* by single primer PCR. The homology of the two TASs is relatively low and with the similarity of 38.90%. Chromosome FISH localization of the two sequences also showed obvious differences in chromosome distribution and signal strength (Fig. 5A, B), which may be due to the differences of chromosome specificity and sequence copy number of the two TASs. In the early study of *Chironomus palidivittatus* (Edwards, 1929) TAS, it was found that there were considerable differences in TAS between species, within species, and even in telomere of the same species (Cohn and Edstriim 1992). Gong et al. cloned six TASs in rice and found high polymorphism of these sequences through RFLP analysis (Gong et al. 1998). From then on, this phenomenon has been found in related studies of other species (Li et al. 2009). Therefore, TASs show great specificity, unlike the more conservative telomere repeated sequences (TR).

Since telomere and adjacent subtelomere regions could not be covered by PAC and BAC clones, sequencing efforts were unable to reveal the structure of these regions. In addition, the discovery of interstitial telomeric sequences (ITSs) makes telomeric minisatellites have double-faced character, which causes more problems in producing genomic assemblies (Richards et al. 1991, Sýkorová et al. 2003). Therefore, nucleotide sequences in the subtelomere regions have not been fully resolved in many genomes that have been sequenced (Mefford and Trask 2002, Mizuno et al. 2006), which greatly affects the quality of genome assembly. FISH localization can reflect the true position of DNA fragments in chromosomes (Jiang and Gill 2006). FISH combined with genomic BLAST can intuitively judge the genomic assembly quality of DNA sequences. Chromosomal locations of 45S rDNA in *G. hirsutum* had been revealed using double-probe FISH, that is, chromosomes A09, D07 and D09 (Gan et al. 2013). In this study, according to the genome BLAST and chromosome FISH localization results of GhTAS and 45S rDNA, it was found that TASs at the end of some chromosomes were not assembled in the genome sequence map. Obviously, results of blastn showed that GhTAS1 was only mapped onto chromosomes D06, D03, A01, D02 and D01 (Table 3, Fig. 4A), but FISH showed more chromosomes carried GhTAS1 signals, including two of the three chromosomes with 45S rDNA A09, D07 or D09, which had not appeared on the blastn results. That is, GhTAS1 sequences may be missed on these assembled chromosomes. The results provide important evidence for the evaluation of the integrity of assembled chromosome end sequences.

## Conclusions

We cloned two telomere-associated sequences from *G. hirsutum* acc. TM-1 using the single-primer PCR, and made analysis about the sequence characteristics of two TASs. The two TASs sequences were enriched in A/T, and were flanked by the forward and inverted primer sequences at each end respectively. By comparative analysis based on the results of blastn and FISH localization of the two TASs, we found that TASs at the end of some chromosomes were not assembled in the genome sequence map. Our study not only contributes to the analysis of telomere structure of cotton, but also provides intuitive evidence for the evaluation of the integrity of the assembled *G. hirsutum* genome.
